# Glycosphingolipids-Dependent Phospholipid Metabolism Enhances Cancer Initiation and Progression through SMPD1/GLTP/B3GALT4/ST8SIA6 Signaling Axis: A Novel Therapeutic Target

**DOI:** 10.7150/ijms.103834

**Published:** 2025-01-06

**Authors:** Liangpan Shi, Nanqi Mao, Zhihua Zheng, Jiangrui Liu, Hao Zhou, Jianbin Hou, Yibin Su

**Affiliations:** Department of Gastrointestinal Surgery, The First Hospital of Quanzhou Affiliated to Fujian Medical University, Quanzhou, 362002, China.

**Keywords:** Colorectal cancer, Phospholipid metabolism, Molecular mechanisms, Single-cell sequencing analysis

## Abstract

Colorectal cancer (CRC) is a prevalent malignancy with high morbidity and mortality rates globally. Advances in single-cell sequencing technology have enabled comprehensive analyses of tumor cells at single-cell resolution, providing valuable insights into the molecular mechanisms underlying CRC initiation and progression. In this study, we integrated single-cell sequencing data with the TCGA database to identify key molecular pathways involved in CRC pathogenesis. Our analysis revealed that dysregulation of phospholipid metabolism, particularly sphingolipid metabolism, plays a crucial role in CRC development. Specifically, we observed aberrant expression of genes involved in sphingolipid biosynthesis and degradation, as well as altered levels of various sphingolipid metabolites in CRC cells. Furthermore, we identified several potential therapeutic targets, including *SMPD1*, *GLTP*, *B3GALT4*, and *ST8SIA6*, within the sphingolipid metabolism pathway that could be exploited for the development of novel CRC treatments. Overall, our findings provide novel insights into the molecular mechanisms underlying CRC and highlight the importance of targeting phospholipid metabolism, specifically sphingolipid metabolism, as a potential therapeutic strategy for CRC.

## Introduction

Cancer remains a major health challenge for humanity, with colon cancer being a common gastrointestinal tumor. Due to anatomical continuity, colon cancer data is often reported together with rectal cancer^1^. In 2020, colorectal cancer ranked third in global cancer incidence and mortality rates were also among the highest. Since the mid-1990s, rectal cancer has exhibited a long and sharp increase, with an annual increase of 1.3% in adults aged 40-49 and 0.5% in adults aged 50-54^2^. The treatment of colorectal cancer mainly involves surgery and chemotherapy, with a significant disparity in 5-year survival rates and prognosis based on different stages. The 5-year survival rate for stage IV colon cancer can be as low as 10%, posing a significant burden on human health and resulting in substantial socio-economic costs^3^. Therefore, there is crucial clinical significance in seeking new screening and treatment methods at an early stage.

The etiology of colorectal cancer is not yet fully understood, and most cases are the result of multiple risk factors acting together. In addition to gender, age, and genetic factors, the influence of lifestyle factors cannot be ignored, such as alcohol consumption, smoking, high-fat and low-fiber diets, and lack of physical activity^4^, ^5^. Various factors disrupt the intestinal microbiota, leading to intestinal permeability and affecting the integrity of the intestinal epithelium. Consequently, they alter intestinal mucosal barrier immunity, reduce nutrient absorption, and regulate secondary bile acid metabolites^6^. Due to the metabolic changes mentioned above, an excess of free radicals derived from secondary bile acid metabolites is produced, leading to an imbalance between endogenous antioxidants and free radicals^7^. The intestinal mucosa and epithelium undergo severe stress responses, resulting in local and even systemic inflammatory reactions and infiltration. All these factors ultimately contribute to various gastrointestinal diseases, including colorectal cancer, inflammatory bowel disease, and gastritis^8^.

In summary, there are many factors contributing to the development of colon cancer, making its pathogenesis highly complex. In recent years, with the continuous optimization and development of genetic engineering techniques, high-throughput sequencing, and single-cell sequencing technologies, a large number of metabolism-related genes associated with colon cancer have been discovered, participating in crucial signaling pathways of colorectal cancer. In 2018, Wataru Sakamoto and his research team found that neutral ceramidase (nCDase), which regulates sphingolipid metabolism, is located in the Golgi apparatus of colon cancer cells and protects cells from ceramide-induced apoptosis^9^. More recently, researchers have identified specific genes, GSTA1, TONSL, and AGA, through a mixed-effect scoring test, confirming their involvement in homocysteine-related amino acid metabolism and folate interaction, thus impacting the occurrence and development of colorectal cancer^10^. Results from a study by Professor Dong Ho Lee and his team at Seoul National University in Korea showed a positive correlation between the total sum of metabolic syndrome (MetS) components and the risk of early-onset distal colon and rectal cancer, but no influence on proximal colon cancer^11^. Based on the researchers' continuous studies in recent years, the treatment of colorectal cancer not only involves early surgical intervention and traditional radiotherapy and chemotherapy but also includes targeted therapy and immunotherapy, which have shown significant efficacy. By combining comprehensive treatments and developing precise personalized treatment plans based on tumor characteristics, we aim to alleviate the physical and economic burdens on patients throughout the disease progression and treatment process. Therefore, in-depth research and exploration of relevant targets involved in the disease development process remain integral to our daily work, providing more possibilities for the diagnosis, treatment, and evaluation of the disease.

## Methods

### Ethical statement

This study was approved by the Ethics Committee of the First Hospital of Quanzhou Affiliated to Fujian Medical University, QuanZhou (No. GSE163974).

### Single-cell sequencing data analysis

To analyze the single-cell sequencing data of colon cancer from GSE163974 using the R language, you can follow these steps: (1) Data downloading and import: Start by downloading the GSE163974 dataset from GEO database (https://www.ncbi.nlm.nih.gov/geo/query/acc.cgi)^12^, and import it into the R environment. The R packages of `GEOquery` and `Seurat` were included for this purpose^13^, ^14^. (2) Data preprocessing: Apply quality control and preprocessing steps to the single-cell sequencing data. This involves removing low-quality cells, low-expressed genes, and outliers. Packages of `Seurat` provide useful functions like `FilterCells()`, `NormalizeData()`, and `ScaleData()` for this step. (3) Data visualization and exploratory analysis: Use scatter plots, box plots, heatmaps, and other visualization techniques to explore the single-cell data. This plot could observed cell distributions, clustering patterns, and differential gene expression. The `Seurat` package offers functions of `DimPlot()` and `FeaturePlot()` for visualization. (4) Cluster analysis: Utilize clustering algorithms to group similar cells together and assign cluster labels. Common clustering algorithms include k-means, DBSCAN, and hierarchical clustering. The `Seurat` package's `FindClusters()` function used for this analysis. (5) Differential analysis: Perform differential expression analysis to identify genes with significant differential expression among different clusters. The `Seurat` package's `FindMarkers()` function were applied. (6) Cell type annotation: Based on the results of differential analysis, annotate the cell types for each cluster using a reference genome or cell type-specific gene set. Tools of `SingleR` package and `Seurat` package's `AddModuleScore()` function used for this purpose^15^. (7) Dynamic analysis and pseudotime trajectory: If your dataset includes time-series measurements, apply dynamic analysis methods and pseudotime analysis algorithms to study the developmental patterns of colon cancer cells. The `Seurat` package provides methods of `Monocle` and `PAGA` for dynamic simulation and pseudotime analysis^14^. Herein, these methods for analyzing the GSE163974 colon cancer single-cell sequencing data using the R language.

### Gene function and pathway enrichment analysis

Gene function and pathway enrichment analysis plays a crucial role in understanding the biological significance of differentially expressed genes. In this methodology, (a) Gene Ontology (GO) Enrichment Analysis: (1) Retrieving GO annotation data: Download and import the required GO annotation files or use pre-existing ones from DAVID databases (https://david.ncifcrf.gov)^16^. (2) Mapping gene identifiers: Use R packages of org.Hs.eg.db to perform mapping and annotation, to ensure that the gene identifiers in the annotation file match those in the expression dataset^17^. (3) Performing GO enrichment analysis: Utilize R packages of clusterProfiler and GOstats to identify significantly enriched GO terms among differentially expressed genes (DEGs). Perform statistical tests of Bonferroni to calculate the enrichment significance of each term with the B-H adjusted p-value less than or equal 0.05. (b) Pathway Enrichment Analysis: (1) Retrieving pathway annotation data: Download or retrieve the relevant pathway annotation files, which can be obtained from KEGG database (https://www.kegg.jp); (2) Mapping gene identifiers: Ensure the gene identifiers in the pathway annotation file match those in the expression dataset. Use R packages of clusterProfiler to perform mapping and annotation^18^. (3) Utilizing statistical approaches of Bonferroni to identify the enriched pathway with the B-H adjusted p-value less than or equal 0.05.

### Colon cancer TCGA database analysis

Here, this methodology outlines the steps involved in analyzing COAD from The Cancer Genome Atlas (TCGA) database using the R language. (1) Accessing TCGA data: Obtain colon cancer data from TCGA through the Genomic Data Commons (GDC) Data Portal or using R package of TCGAbiolinks^19^, ^20^. Download relevant data files^21^, including gene expression and clinical information^22^. (2) Data Preprocessing: (a) Data quality control: Perform quality assessment and filtering of the TCGA datasets to ensure the removal of low-quality or unreliable samples. Implement quality control checks based on specific criteria, such as sample integrity, sequencing depth, or batch effects. (b) Gene expression normalization: Apply normalization techniques of DESeq2, to account for variation in gene expression due to technical biases. This step harmonizes gene expression values for subsequent analysis. (3) Differential Expression Analysis: utilize R packages of DESeq2 to identify DEGs between COAD samples and normal controls. Adjust for multiple testing to control for false discovery rate (FDR). The statistical thresholds to determine significant DEGs based on log2|fold change| over than or equal 2 and B-H adjusted p-value less than or equal 0.05.

### Gene Set Enrichment Analysis (GSEA) and Gene Set Variation Analysis (GSVA)

GSEA and GSVA are powerful tools for identifying biological pathways or gene sets that are differentially regulated in various experimental conditions^17^, ^23^-^26^. This methodology provides a comprehensive guide for conducting GSEA and GSVA analysis to gain insights into pathway level changes in gene expression^27^-^30^. Gene sets were obtained from MSigDB databases (https://www.gsea-msigdb.org/gsea/index.jsp). Here, the analysis as following: (a) Preranked gene list: Rank genes based on their differential expression between different conditions or phenotypes. Use statistical methods of limma to compute fold changes and p-values. (b) Running GSEA: Utilize R packages of clusterProfiler, fgsea, and GSEABase to perform GSEA analysis. Input the ranked gene list and the gene set collection. Generate an enrichment score (ES) and a normalized enrichment score (NES) to determine the significance of gene set enrichment. (c)Visualize GSEA results using enrichment plots, showing the ranking of genes along with running enrichment scores. Identify significantly enriched gene sets based on the NES and assess their biological relevance.

For the GSVA analysis, the following steps were applied: (1) Gather the gene sets that represent biological pathways, processes, and functional gene categories. These gene sets also can be obtained from MSigDB databases^28^, ^29^. (2) GSVA calculation: Use R packages of GSVA to perform GSVA analysis, including the steps of (a) Input the gene expression matrix and the gene set collection; (b) Compute pathway enrichment scores for each sample, representing the activity level of each pathway^31^. (3) Differential GSVA analysis: Compare the pathway enrichment scores between different conditions or phenotypes. Use statistical methods of limma analysis to identify significantly differentially enriched pathways. (4) Visualize GSVA results using heatmaps and bar plots, showing the pathway enrichment scores for each sample. Assess the differences in pathway activity levels between conditions.

### Survival analysis

Here, the hub genes and pathway enriched score survival analysis applied as following^32^, ^33^: (1) overall survival (OS) analysis: Assess the relationship between gene expression and patient survival. Utilize R packages including survival, survminer, and survivalROC for survival analysis and generate Kaplan-Meier curves. (2) Cox proportional hazards regression: Perform Cox regression analysis to evaluate the prognostic value of DEGs. Identify genes that significantly impact patient survival using R packages of survival and survminer^32^.

## Results

### Single-cell sequencing data analysis

**Figure 1:**
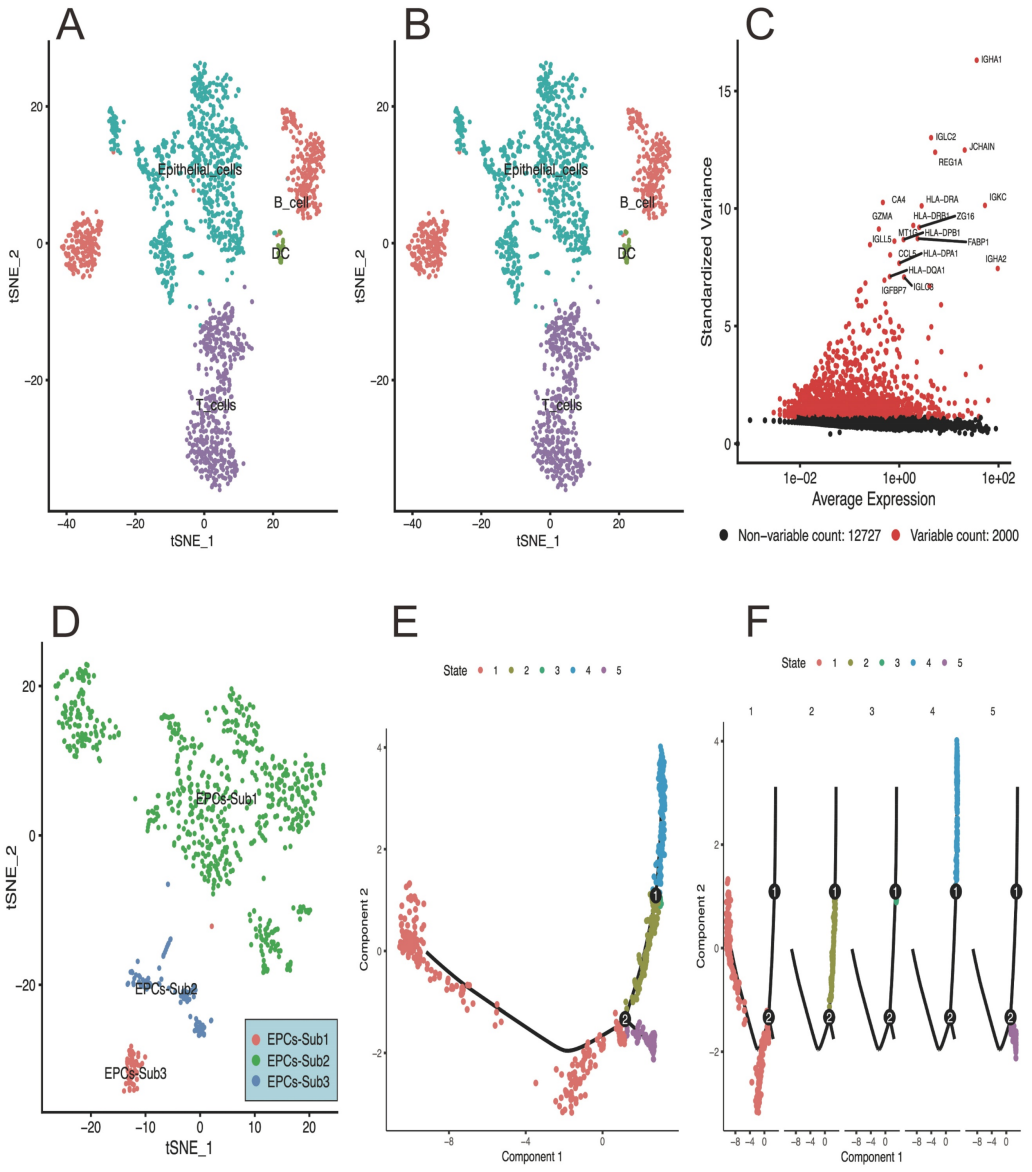
** The results of single-cell sequencing data analysis in response to CRC development.** A) the PCA plot represents the clustering pattern of cells based on their gene expression profiles, and shows the distribution of cells in a reduced-dimensional space, with each point indicating an individual cell. B) showing the cells relationship by projecting high-dimensional gene expression data into a two-dimensional space. C) highlights the expression level of specific genes across different cell populations or clusters. D) presents the distribution of epithelial cells in a reduced-dimensional space, And E and F showing the pseudotime inference algorithm assigns a pseudotime value to each cell, allowing the assessment of temporal relationships and differentiation paths.

Based on the single-cell RNA-sequencing analysis, the results can be summarized as follows: Figure 1A of the PCA plot represents the clustering pattern of cells based on their gene expression profiles. It shows the distribution of cells in a reduced-dimensional space, with each point indicating an individual cell. Clusters of cells that exhibit similar gene expression profiles tend to be closer together, while cells with distinct expression patterns are located farther apart. Figure 1B visualizes the relationship between cells by projecting high-dimensional gene expression data into a two-dimensional space. Cells that are more similar in terms of gene expression are placed closer together in the plot, and providing information about cell type heterogeneity and allows for the identification of distinct subpopulations within the dataset. This plot of Figure 1C highlights the expression level of specific genes across different cell populations or clusters. It displays the enrichment or depletion pattern of selected genes in a group-wise or cell type-specific manner. This information is helpful for identifying genes that are differentially expressed and potentially associated with specific cellular functions or biological processes. Figure 1D of the tSNE map presents the distribution of epithelial cells in a reduced-dimensional space, representing distinct subpopulations with unique spatial relationships. And figure 1E-F provides insights into the pseudotime-based progression of EPCs subpopulations.

### Gene Function and Pathway Enrichment Analysis

**Figure 2:**
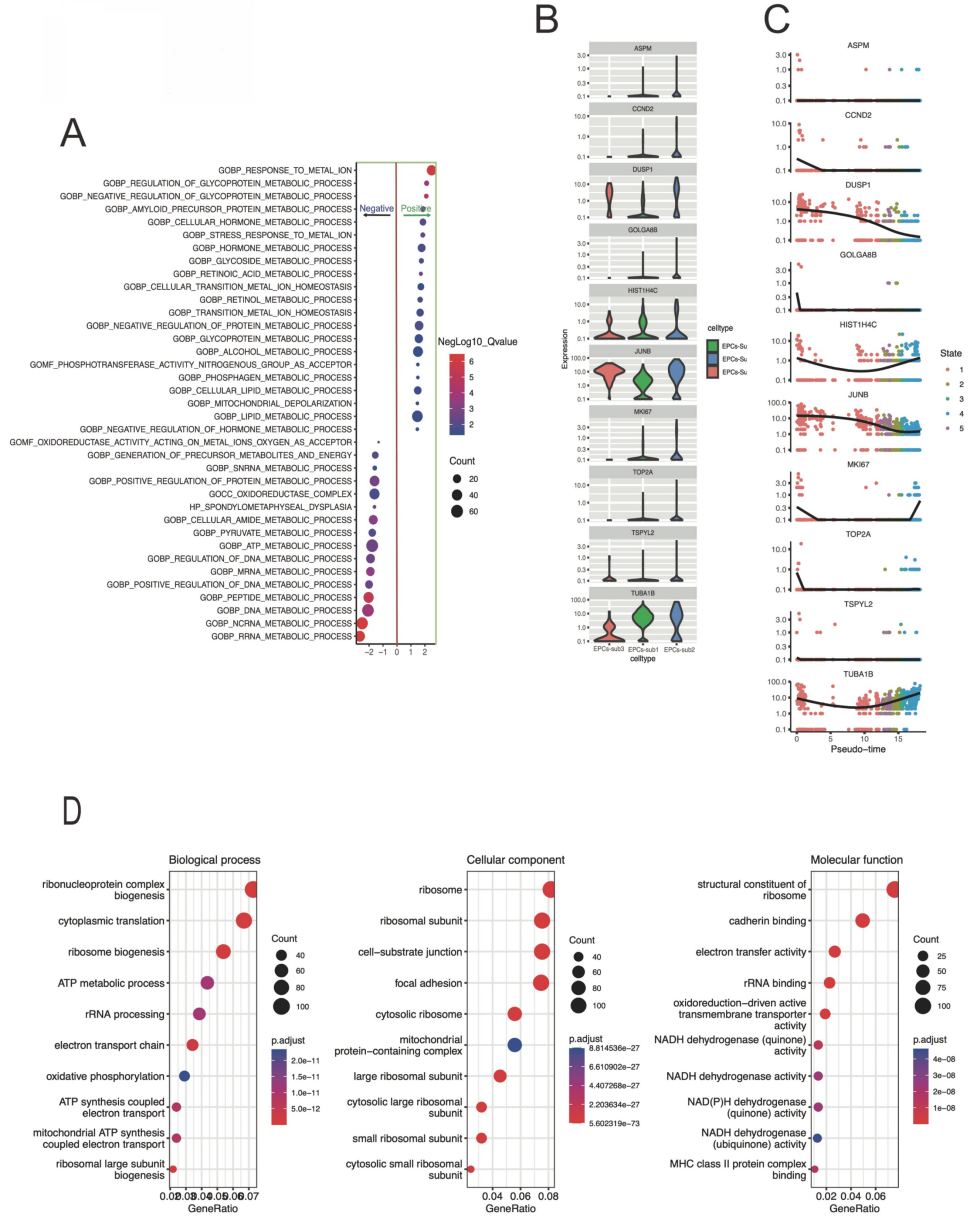
** The gene functional enrichment results in response to pseudotime-related differently expressed genes.** A) The bubble plot indicating the enrichment level and significance of the gene set under different conditions. The bubbles placed closer to the top of the plot indicate higher enrichment levels, and the size of the bubbles represents the enriched size. While x-axis representing the significance of the enrichment. B-C) the box and dot-plot presenting the expression level differences of core genes in different cell EPCs-subtypes. D) the bubble plot represents the specific GO term.

The GSEA bubble plot of Figure 2A demonstrates the enrichment level and significance of gene sets under different conditions. Here, GOBP: response to metal ion, GOBP: regulation of glycoprotein metabolic process, and GOBP: negative regul ation of glycoprotein metabolic process were positivetlly enriched. While the GOBP: DNA metabolic process, GOBP: ncRNA metabolic process, and GOBP: RRNA metabolic process were negativetlly enriched. And, clearly, metabolic related signaling pathways are significantly enriched. Figure 2B-C presents the distribution of hub gene expression levels in different subtypes. The figure 2D showing the result of a GO analysis, and the bubble plot represents a specific gene ontology term involved in biological process, molecular function, or cellular component, which provides a visual representation of enriched gene ontology terms. And the terms of ATP metabolic process, mitochondrial ATP synthesis, and oxidative phosphorylation were significantly enriched.

**Figure 3:**
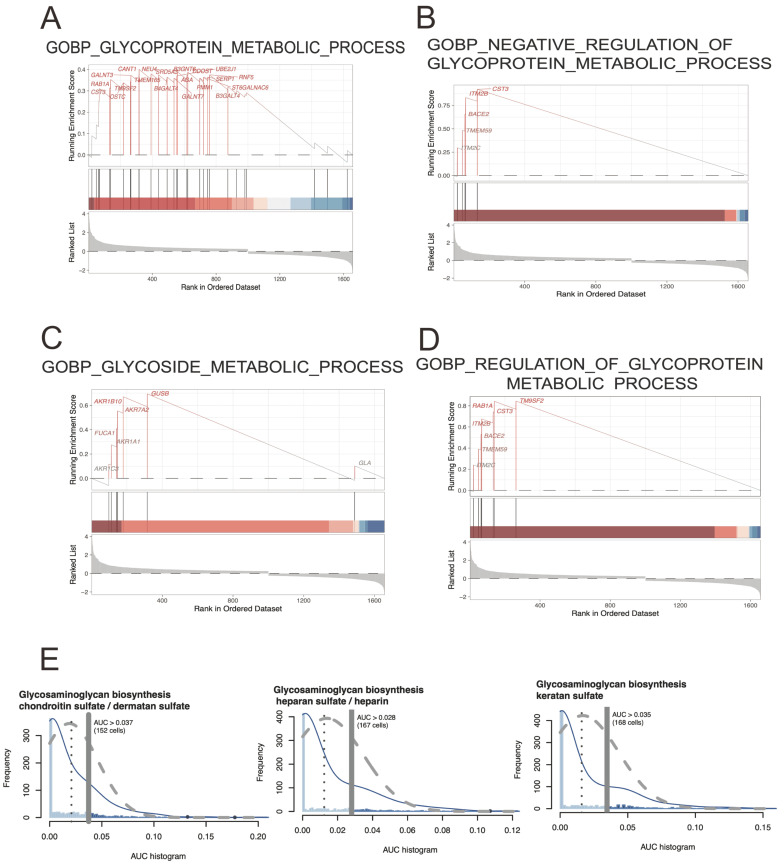
** The analysis of GSEA and AUC-based scoring of cell metabolic pathways.** A-D) representing the GSEA enriched results involved in glycoprotein metabolic process. E) showing the implementation of an AUC-based scoring approach allows for the quantification of cellular metabolic pathway activity using single-cell sequencing data of EPCs.

The result of a GSEA analysis typically includes a ranked list of genes, with the most strongly correlated genes at the top of the list. This list is then compared against a collection of predefined gene sets, which represent known biological pathways, molecular functions, and cellular processes. And terms including GOBP: glycoprotein metabolic process (ES=0.40; NES=1.56; p-adjusted=0.04), GOBP: negative regulation of glycoprotein metabolic process (ES=0.92; NES=2.10; p-adjusted=1.87E-05), GOBP: glycoside metabolic process(ES=0.69; NES=1.75; p-adjusted=0.019), and GOBP: regulation of glycoprotein metabolic process (ES=0.84; NES=2.13; p-adjusted=2.31E-04) were significantly detected. Here, the map of glycoprotein metabolic process may play an important role in CRC development. The figure 3E showing the Area Under the Curve (AUC)-based scoring of cell metabolic pathways involved in glycoprotein metabolic process, and showing the cell heterogeneity of glycoprotein metabolic pathway activity within EPCs cell population and detecting the regulatory mechanisms underlying cell metabolism.

### Colon Cancer TCGA Database Analysis

**Figure 4:**
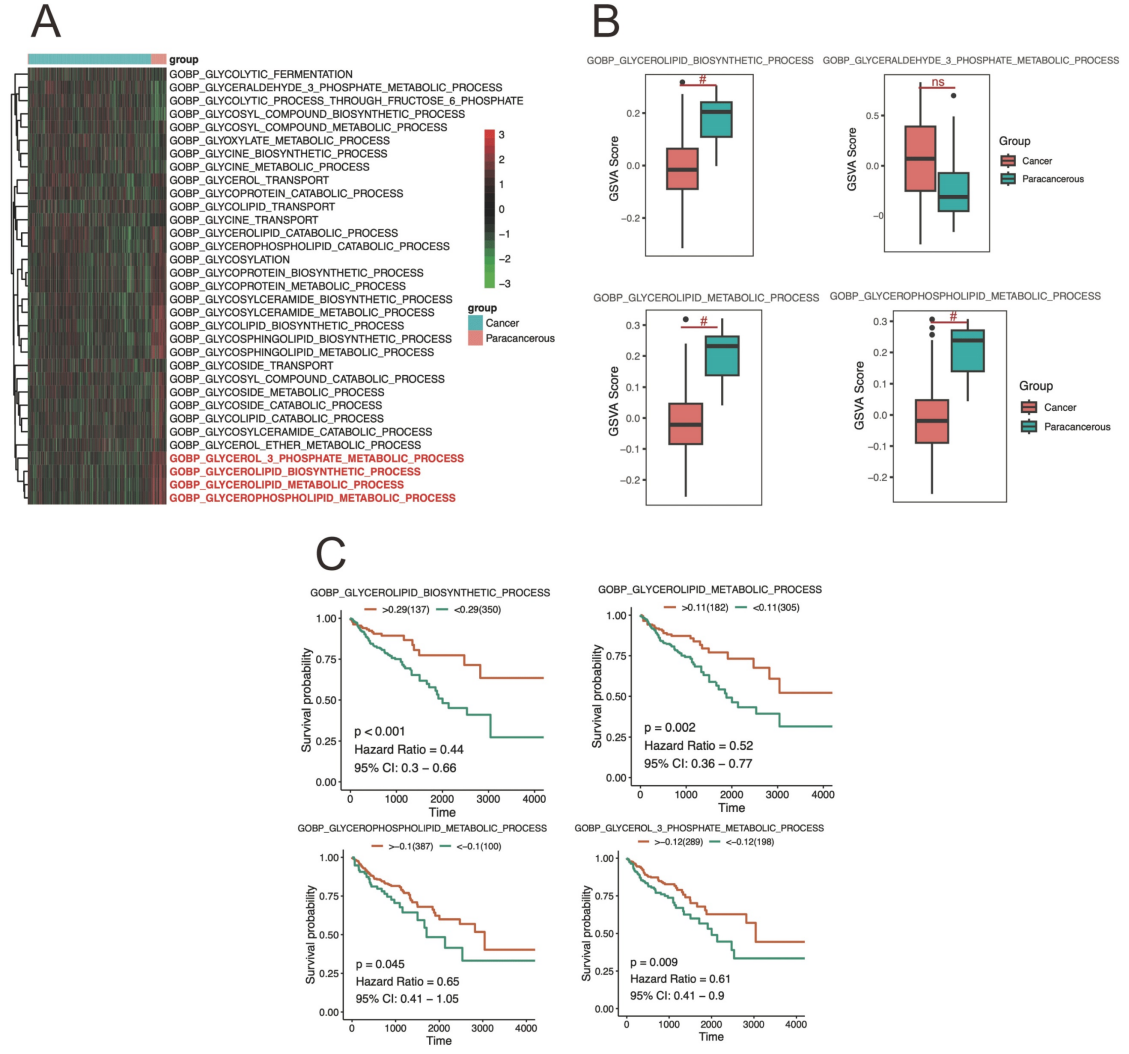
** The differently level and survival analysis of CRC cancer tissue from TCGA.** A-B) showing the results of differential enrichment scores of glycoprotein metabolic GSVA pathways in CRC cancer tissue. C presented the glycoprotein metabolic alterations associated with CRC cancer development and progression based on survival analysis.

GSVA measures the enrichment of gene sets within a sample, providing an indication of pathway activity. And the differences in enrichment scores of metabolic pathways related to CRC tissue were analyzed using the GSVA method in Figure 4A-B. Here, the GSVA was employed to calculate the enrichment scores for metabolic pathways, reflecting their relative activity levels in CRC sample (Figure 4A). A differential analysis was then performed to compare the enrichment scores between different groups in figure 4B. The results revealed significant differences in the enrichment scores of glycoprotein metabolic process among the cancer and paracancerous tissues, and suggest alterations in the activity levels of glycoprotein metabolic pathways in CRC cancer tissue compared to healthy tissue.

The analysis showed that glycoprotein metabolic pathways were significantly correlated with CRC patient survival. Here, high activity levels in the GOBP: glycoprotein metabolic, GOBP: negative regulation of glycoprotein metabolic process, GOBP: glycoside metabolic process, and GOBP: regulation of glycoprotein metabolic process were not favorable to overall survival, suggesting that the activation of this pathway may have a exacerbate damage for CRC progression (Figure 4C). These findings highlight the potential prognostic value of pathway activity levels in CRC cancer and provide insights into the molecular mechanisms underlying cancer progression. Further investigations are warranted to validate these results and explore the therapeutic implications of targeting specific pathways in the treatment of CRC cancer.

### Hub regulator detection

**Figure 5:**
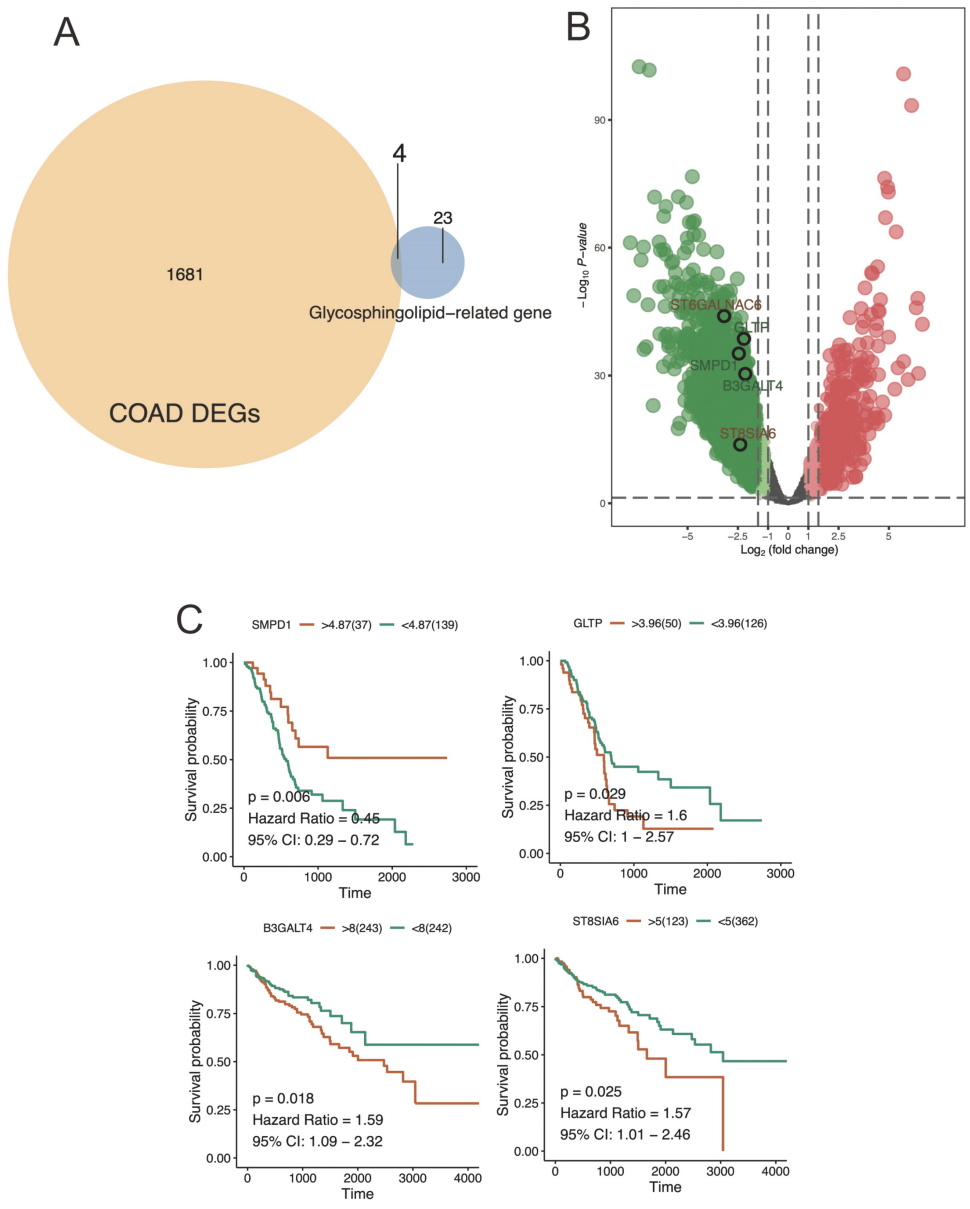
** Venn diagram and volcano plot were used to illustrate the results of hub gene analysis.** A) This venn plot provided a clear visual representation of the shared and unique core genes across TCGA-based COAD DEGs and glycosphingolipid-related gene. Each circle in the diagram represented a different condition or group, and the intersection of circles represented the common genes found in those conditions or groups. B) The hub genes exhibiting a significant fold change with a high statistical significance were located further away from the center of the plot, resembling the shape of a volcano. C) the survival analysis of key regulators expression level revealed significant associations between gene expression levels and patient survival outcomes.

The venn diagram displayed the overlap of core genes identified from TCGA-based COAD DEGs and glycosphingolipid-related gene (Figure 5A). and the volcano plot, on the other hand, presented a representation of the statistical significance and fold change of hub gene expression between cancer and paracancerous tissue (Figure 5B). Similarly, the key regulators including *SMPD1*, *GLTP*, *B3GALT4*, and *ST8SIA6* were significantly correlated with CRC patient survival. Here, high expression levels in *SMPD1*, and low expression in *GLTP*, *B3GALT4*, and *ST8SIA6* were significantly correlated with worse prognosis, suggesting a significant relations to *SMPD1*, *GLTP*, *B3GALT4*, and *ST8SIA6* expression level of the development and prognoses of the CRC patients (Figure 5C).

## Discussion

In this study, we conducted an analysis and screening of potential core pathways, specifically the Glycosphingolipid pathway, and identified four core genes (*SMPD1*, *GLTP*, *B3GALT4*, and *ST8SIA6*) in colorectal cancer based on data analysis from relevant databases. We also demonstrated significant differential expression of these core genes through modeling. Lipid metabolism dysregulation is one characteristic of cancer cells. Highly proliferating cancer cells not only require lipids for cell membrane synthesis (phospholipids, cholesterol, and sphingolipids) but also utilize lipids as substrates for energy metabolism (triglycerides) or as sources of signaling molecules. Bioinformatics analysis and different analytical methods applied to clinical samples have shown significant changes in the species profile of sphingomyelins (SM) and triglycerides (TG) in colorectal cancer cohorts. These changes were successfully validated in two independent cohorts and showed a significant correlation with postoperative survival rate, supporting the hypothesized clinical relevance. The dysregulation of glycerolipid metabolism mentioned in this study may be involved in the progression of CRC, with the core genes *SMPD1*, *GLTP*, *B3GALT4*, and *ST8SIA6* playing crucial roles.

Glycosphingolipids (GSLs) are a class of complex glycoconjugates that play crucial roles in various cellular processes, including signal transduction, cell adhesion, and modulation of immune responses^34^-^36^. And GPLs are composed of a hydrophobic ceramide domain linked to a hydrophilic carbohydrate moiety. They are broadly classified into ganglio-series, globo-series, isoglobo-series, and lacto/neo-lacto-series based on their carbohydrate structures^34^, ^37^. Dysregulation of GSL metabolism can occur through altered expression or activity of enzymes involved in GSL biosynthesis, degradation, and modification^38^. Accumulating evidence suggests that aberrant GSL metabolism contributes to tumor initiation, progression, metastasis, and drug resistance^37^, ^39^. Studies have demonstrated that alterations in GSL metabolism can directly impact oncogenic signaling pathways. For example, aberrant expression of glycosyltransferases involved in GSL synthesis has been observed in various cancers, leading to changes in GSL composition and functions^35^, ^39^, ^40^. Altered GSL expression can modulate key cellular events, such as cell proliferation, apoptosis, invasion, and angiogenesis, thereby promoting tumor initiation and growth. Several studies have reported the association between dysregulated GSL metabolism and tumor development. For instance, increased expression of specific GSLs, such as GM2 and GD2, has been observed in neuroblastoma and melanoma, respectively, and is associated with poor prognosis^34^. Moreover, GSLs have been implicated in cancer stem cell maintenance and epithelial-mesenchymal transition, both of which are critical processes in tumor development and metastasis^35^. Targeting dysregulated GSL metabolism shows promise as a therapeutic strategy for cancer treatment. Inhibition of GSL synthesis enzymes, such as glucosylceramide synthase, has been explored as a potential anticancer therapy^35^. Additionally, the development of antibodies targeting GSL antigens, such as GD2, has shown encouraging results in clinical trials, highlighting the therapeutic potential of targeting GSLs^41^. Here, the dysregulation of glycosphingolipid metabolism is implicated in the occurrence and development of various types of tumors. Aberrant GSL expression and altered GSL-dependent signaling pathways contribute to tumor initiation, progression, and metastasis^37^. Further investigations into the precise mechanisms underlying the relationship between dysregulated GSL metabolism and cancer are warranted to exploit GSL-related targets for novel therapeutic interventions in cancer treatment^42^.

Researchers have indicated that 5-fluorouracil (5-FU)-resistant colon cancer cells predominantly acquire resistance by inhibiting cell apoptosis induced by ceramide through the sphingomyelin (SM)/ceramide pathway, with lower levels of *SMPD1*^43^-^45^, compared to sensitive cell lines. *SMPD1*translocates to the plasma membrane to generate ceramide, promoting enhanced apoptosis signaling through FAS-FASL and TNFRSF10-TNFSF10, leading to rapid endothelial cell apoptosis. In early investigations, by comparing *SMPD1* activity and tumor cell apoptosis rates, the appropriate radiation dosage for CRC radiotherapy could be selected^43^-^45^. And these results highlighted the upregulation of MIR196B in CRC tissues, which regulates the expression levels of GLTP during the colorectal cancer (CRC) development process^46^-^48^. In human colon cancer cells, GLTP overexpression interferes with cell cycle progression, induces cell death, and inhibits cell growth^49^. Recent studies have discovered that *GLTP*, as a differentially expressed death-related gene, is associated with poor prognosis in female cervical cancer^46^-^48^. Zhang *et al.* found that overexpression of the *B3GALT4* glycosyltransferase responsible for ganglioside GM1 synthesis can induce epithelial-mesenchymal transition (EMT) in breast cancer cells^50^. Additionally, analysis shows that *B3GALT4* can independently predict overall survival in osteosarcoma patients, suggesting its potential as a prognostic biomarker^15^, ^49^, ^51^. Early studies demonstrated that downregulation of *B3GALT4* in neuroblastoma cells resulted in increased proliferation, invasion, and metastasis abilities *in vitro*^21^, ^31^, ^50^, ^52^-^59^. High expression of ST8SIA6-AS1 was detected in hepatocellular carcinoma tissues and cells, with ST8SIA6-AS1 silencing leading to weakened proliferation and migration abilities in liver cancer cells^60^, ^61^. ST8SIA6-AS1 displayed its oncogenic function through the absorption of the tumor suppressor miR-651-5p. Shih *et al.* showed that ST8SIA6-silenced colon cancer cells exhibited increased resistance to treatment with ibrutinib^42^, ^51^, ^62^, as shown by cell viability assays^60^, ^61^, ^63^-^68^. Moreover, downregulation of *ST8SIA6* was positively correlated with cancer recurrence in later stages. *ST8SIA6* accelerated tumor occurrence in a genetically engineered spontaneous mouse model of colon cancer, reducing survival to approximately 67 days^60^, ^61^, ^69^-^72^. Thus, *ST8SIA6* expression in tumors suppresses anti-tumor immune responses to enhance tumor growth^22^, ^33^, ^73^. However, this study also has limitations and drawbacks, such as the lack of large sample clinical cohort studies and experimental validation.
